# A study on the correlation between pain frequency and severity and vitamin B12 levels in episodic and chronic migraine

**DOI:** 10.1590/0004-282X-ANP-2021-0192

**Published:** 2022-08-08

**Authors:** Sibel Üstün Özek

**Affiliations:** 1 University of Health Sciences, Prof. Dr. Cemil Taşçıoğlu City Hospital, Department of Neurology, Istanbul, Turkey

**Keywords:** Migraine Disorders, Headache, Vitamin B 12, Homocysteine, Transtornos de Enxaqueca, Cefaleia, Vitamina B 12, Homocisteína

## Abstract

**Background:**

It is believed that vitamin B12 deficiency and hyperhomocysteinemia cause endothelial cell damage by increasing the levels of free oxygen radicals, which may, in turn, be related to the onset of migraine episodes.

**Objective:**

The objective of our study was to ascertain a correlation between vitamin B12 levels and migraine attack frequency and pain severity.

**Methods:**

127 patients with migraine and 45 healthy controls who presented to Okmeydanı Training and Research Hospital were included in the study. The migraine attack frequency and the duration and severity of pain in migraineurs were recorded. Pain severity was evaluated using a visual analogue scale (VAS). Vitamin B12 levels below 300 ng/L were considered low.

**Results:**

The vitamin B12 levels in migraineurs were found to be significantly lower than those in the control group (227.30 ± 104.72 ng/L vs 278.44 ± 149.83 ng/L; p = 0.047). The vitamin B12 levels of patients with chronic migraine (CM) were found to be lower than those in patients with less frequent migraine attacks (197.50 ± 69.16 ng/L vs 278.56 ± 147.91 ng/L; p = 0.019). The ratio of vitamin B12 levels of 300 ng/L and above in patients with CM was lower than that of patients with episodic migraine (p < 0.05).

**Conclusions:**

Along with attack frequency and pain severity assessment, it is important that migraine follow-ups should include regular measurement of vitamin B12 levels. We found lower vitamin B12 values in the CM group.

## RESUMO

**Antecedentes:**

Acredita-se que a deficiência de vitamina B12 e a hiper-homocisteinemia causem danos às células endoteliais pelo aumento dos níveis de radicais livres de oxigênio, o que pode, por sua vez, estar relacionado ao aparecimento de episódios de enxaqueca.

**Objetivo:**

O objetivo do nosso estudo foi verificar a correlação entre os níveis de vitamina B12 e a frequência e a gravidade da dor nas crises de enxaqueca.

**Métodos:**

127 pacientes com enxaqueca e 45 controles saudáveis ​​que se apresentaram ao Okmeydanı Training and Research Hospital foram incluídos no estudo. A frequência das crises de enxaqueca, bem como a duração e a gravidade da dor nos pacientes foram registradas. A gravidade da dor foi avaliada usando-se uma escala visual analógica (EVA). Níveis de vitamina B12 abaixo de 300 ng/L foram considerados baixos.

**Resultados:**

Os níveis de vitamina B12 em pacientes com enxaqueca foram significativamente menores do que os do grupo controle (227,30 ± 104,72 ng/L vs 278,44 ± 149,83 ng/L; p = 0,047). Os níveis de vitamina B12 de pacientes com enxaqueca crônica (EC) foram menores do que aqueles em pacientes com crises de enxaqueca menos frequentes (197,50 ± 69,16 ng/L vs 278,56 ± 147,91 ng/L; p = 0,019). A proporção dos níveis de vitamina B12 de 300 ng/L e acima em pacientes com EC foi menor do que a de pacientes com enxaqueca episódica (p < 0,05).

**Conclusões:**

Juntamente com a avaliação da frequência das crises e da gravidade da dor, é importante que o acompanhamento da enxaqueca inclua a medição regular dos níveis de vitamina B12, pois encontramos valores mais baixos de vitamina B12 no grupo EC.

## INTRODUCTION

Migraine is a condition that accounts for large patient volumes in daily neurology polyclinics. It causes obstacles to daily life activities and has an adverse impact on quality of life. The ratio of disability and workforce loss increases in parallel with an increase in the frequency and severity of pain^1^. The risk factors regarding migraine that cannot be changed are age, sex and family history. Other risk factors can be changed, and the most common of these are diet, stress, sleep changes, depression, medications and habits like alcohol consumption and caffeine intake^2^. Supplement intake is also considered to be a changeable factor^3^ ^,^ ^4^_._

Patients are diagnosed as having chronic migraine (CM) if pain occurs at a frequency of over 15 days a month, among which a minimum 8 days of pain have the characteristics of migraine^5^. Although transformation from episodic migraine is mostly blamed, it is considered that individuals’ genetic predisposition and certain modifiable or non-modifiable risk factors play a role in this transformation. Although many factors are blamed for migraine becoming chronic and for increasing pain frequency, the underlying pathophysiological mechanisms are not yet altogether clear^6^. Identifying the factors that trigger migraine attacks is important for both diagnosis and preventive treatments. Therefore, modifiable factors need to be evaluated well, and the requirement for nutritional supplements need to be considered.

It is believed that vitamin B12 deficiency and hyperhomocysteinemia cause endothelial cell damage by increasing the levels of free oxygen radicals, which may, in turn, be related to the onset of migraine episodes. It is also believed that nitric oxide (NO) plays a role in the pathology of migraine^7^ ^,^ ^8^. However, vitamin B12 has an attenuating effect on NO^9^_._

There are many studies assessing migraine in relation to vitamin B12 levels. Some found normal levels of vitamin B12, whereas others found low levels in migraineurs^10^ ^-^ ^12^. In the study of İpekçioğlu et al., the level of vitamin B12 was found to be normal in serum, whereas the level of methylmalonic acid, which is a metabolite of vitamin B12, was found to be high in urine^13^. In other studies, apart from assessing vitamin B12 serum levels in migraine with aura and migraine without aura, vitamin B12 serum levels were assessed during the migraine attack and outside of the migraine attack^14^. There are many open-label and randomized control trial studies assessing prophylactic vitamin B12 and replacement treatments^15^. 

When browsing the literature, we found more studies concerning migraine with aura than migraine without aura, in correlation with vitamin B12 levels. These were mostly related to use of vitamin B12 in treatments. In studies concerning migraine with aura that were conducted among children and adolescents^16^ ^-^ ^18^, vitamin B12 replacement was correlated with decreased pain frequency and intensity, along with decreased disability related to migraine. In a study that included migraine cases both with and without aura, pain frequency and use of drugs related to migraine diminished with vitamin B12 replacement, but there was no difference in the duration of attacks^9^. 

Even if there are studies proving the benefit of vitamin B12 replacement in cases of migraine with aura, there is a need for more randomized controlled trials regarding migraine without aura^19^. Vitamin B12 levels were found to be low in many studies concerning pediatric and adult groups of migraineurs^12^ ^,^ ^20^. In those studies, no evaluation of the frequency of attacks was conducted. In one study, evaluations was made during attacks and outside of attacks, and it was found that vitamin B12 levels were lower during the attacks. In one study evaluating vitamin B12 and MMA levels, there was no difference in levels between the episodic migraine group and the chronic migraine group. However, there was higher frequency of migraine among participants with low vitamin B12 levels^21^.

Our study is valuable in these terms, in that it assesses vitamin B12 levels in correlation with pain frequency, mostly in a group of migraineurs without aura. The objective of our study was to ascertain a correlation between vitamin B12 levels and migraine attack frequency and pain severity.

## METHODS

Patients who were diagnosed as having migraine according to the International Classification of Headache Disorders (ICHD-III), from among outpatients presenting to the Okmeydanı Training and Research Hospital neurology polyclinic between 2019 and 2020, were included in this study. Only patients who had complete medical data recordings and in whom vitamin B12 levels were assessed, were retrospectively scanned and included. Those with systemic diseases such as high blood pressure and diabetes mellitus, and those using vitamin replacements, were excluded. Clinical and demographic data such as age at disease onset, duration and frequency of headaches, clinical characteristics and location of the headaches, pain severity, triggering factors, presence of family history, presence of aura and visual analogue scale (VAS) scores were recorded. 

The patients were divided into three groups according to attack frequency. These groups were identified as infrequent episodic, frequent episodic and chronic. Those with 1-3 migraine attacks per month and those having pain on 4-14 days per month were included in the infrequent and frequent episodic groups, respectively. CM was identified as headache attacks lasting over 4 hours on 15 or more days per month for a minimum period of three months, when the attacks on a minimum of eight of these days met the criteria for the diagnosis of migraine. 

The control group consisted of patients who presented to our neurology outpatient clinic for general medical examination, and who were registered with the code Z00.0. Z00.0 is the code used to designate a group of patients who came in for ‘general examination’, who were not diagnosed as presenting any neurological disease and in whom no abnormal symptom was found. These patients had no headache symptoms. Their neurological, imaging and neurophysiological examinations were normal. Patients who had no additional systemic disease were chosen to form the control group. These patients had come in with non-specific complaints and their vitamin B12 assays was made within the context of a general check-up.

Pain severities were evaluated using VAS scores. From among the forms used in the interviews, a VAS was used by patients to subjectively score their pain severity on a horizontal or vertical 10-cm straight-line scale from 0 = no pain to 10 = most severe pain^22^ ^23^.

All the samples from the patients who were diagnosed with episodic and chronic migraine in accordance with the migraine diagnosis criteria were collected during an attack-free period. In our clinic, routine biochemical tests, along with the frequency and severity of pain are evaluated annually or biannually among patients diagnosed with migraine. The routine biochemical tests performed included renal and liver function tests, electrolyte values, hemogram, thyroid function tests, vitamin B12 and folate values. Samples were taken at the time of the first interview and only at this one time. 

The vitamin B12 assays were done as follows: After 12 hours of fasting, 5 mL of blood was taken into a yellow serum separation gel tube. The samples were immediately centrifugated at +4 °C and 4000 rpm for 10 minutes. Vitamin B12 levels were assayed using a chemiluminescent immunoassay in a Roche Cobas Integra 400 Plus analyzer. Levels of below 300 ng/L were considered low for vitamin B12. The groups formed according to migraine frequency were compared with regard to vitamin B12 levels among each other and with normal healthy individuals.

Written informed consent was obtained from all participants in the study. Approval for the study was obtained from the Istanbul Prof. Dr. Cemil Taşçıoğlu City Hospital Clinical Research Ethics Board (Approval No. 32, dated February 9, 2021). This date is different from the selection data. Was the ethics send later? 

### Statistical analysis

The Number Cruncher Statistical System (NCSS) (Kaysville, Utah, USA) software was used for statistical analyses. Complementary statistical methods (mean, standard deviation, median, frequency, ratio, minimum and maximum) were used when evaluating the study data. The suitability of quantitative data for normal distribution was tested using the Kolmogorov-Smirnov test, Shapiro-Wilk test and graphic evaluations. Student’s t test was used in two-group comparisons of quantitative data with normal distribution, and the Mann-Whitney U test was used in two-group comparisons of data without normal distribution. The Kruskal-Wallis test was used for comparing three or more groups that did not demonstrate normal distribution, and the Bonferroni-Dunn test was used for paired comparisons. Pearson’s chi-square test and Fisher’s exact test were used for comparing qualitative data. Binary logistic regression analyses were used. The significance level was taken to be a minimum of p < 0.05.

## RESULTS

There were 127 patients with a diagnosis of migraine (121 females and six males); and 45 controls (43 females and two males) were included in the study. Patients with antecedents of chronic disease or who were using food supplements or any other drug were excluded from the study. The percentage of patients who were excluded was about 16%. None of the patients initially included in the study were lost later on. The mean ages of the patients with migraine and the control group were 37.6 ± 9.7 years and 42.3 ± 10 years, respectively. When gender and age were compared between the study and control groups, the p values detected were p = 0.121 and p = 0.012 respectively. The mean ages of the migraineur groups were 36.83 ± 2.1 in the infrequent episodic group, 35.65 ± 1.32 in the frequent episodic group and 40.14 ± 1.33 in the chronic group. When the migraineur groups were compared with each other, p = 0.059 was found and there was no significant difference according to the age.

The clinical features of migraine are shown in Table 1. Evaluation of the migraine demographic characteristics using the Pearson chi-square test after subtracting the cases of migraineurs with aura showed the following results: phonophobia p = 0.100, photophobia p = 0.107, nausea p = 0.177, vomiting p = 0.599 and family history p = 0.299. These data were comparable to those found from evaluating the group of migraineurs with and without aura all together. The Kruskal-Wallis test showed a statistically significant difference in comparing nonparametric distribution of disease duration and frequency of pain (between the infrequent episodic, frequent episodic and chronic migraine groups) (p = 0.006). Subgroup analysis showed that this difference was specifically between the frequent episodic and chronic groups (p = 0.015).

The mean vitamin B12 level of the study group was 240.68 ± 119.85 ng/L (range, 85-836). The vitamin B12 levels were ≥ 300 ng/L in 25.6% (n = 44) and < 300 ng/L in 74.4% (n = 128). The vitamin B12 levels in migraineurs were found to be significantly lower than in the control group (227.30 ± 104.72 ng/L vs 278.44 ± 149.83 ng/L; p = 0.047); and the vitamin B12 levels of those in the migraine group were lower than those of the control group. The ratio of encountering a vitamin B12 level over 300 ng/L was lower for the migraine group than for the control group (p = 0.029). Comparison of the duration of the disease and vitamin B12 levels using the Mann-Whitney test did not show any statistically significant difference (p = 0.172).

The Pearson chi-square test was used to compare pain frequencies according to vitamin B12 levels (below and above 300), in three groups of migraineurs: infrequent episodic, frequent episodic and chronic migraine. A statistically significant difference was found (p = 0.044). The infrequent episodic and frequent episodic groups were considered as one group because of the low number of cases. Comparison of the episodic pain and chronic pain groups showed that there was a significant difference (p = 0.012) between them. When the cases of migraine with aura were taken out, there was also a statistically significant difference (p = 0.019) (Table 1).

**Table 1.** Demographic characteristics and B12 levels according to migraine frequencies.

		Migraine frequency			p
		Infrequent episodic (n = 18)	Frequent episodic (n = 59)	Chronic (n = 50)	
Vitamin B12		182-836 (239.5)	90-471 (214)	85-406 (184.5)	^e^0.020*
		278.56 ± 147.91	236.92 ± 108.14	197.50 ± 69.16	
Vitamin B12 level; n (%)	< 300	13 (72.2)	42 (71.2)	45 (90.0)	^d^0.044*
	≥ 300	5 (27.8)	17 (28.8)	5 (10.0)	
Disease duration (years)	Min-Max (median)	1-30 (8)	1-38 (6)	1-43 (10)	^e^0.006^**^
	Mean ± SD	10.50 ± 2.13	8.45 ± 1.10	13.42 ± 1.33	
Aura	None Yes	16 (88.8) 2 (11.2)	53 (89.8) 6 (10.2)	50 (100) 0 (0)	^d^0.062
VAS	Min-Max (median)	5-10 (9)	5-10 (9)	7-10 (10)	^e^0.004**
	Mean ± SD	8.67 ± 1.71	8.68 ± 1.18	9.38 ± 0.88	
Family history; n (%)	None	6 (33.3)	28 (47.5)	23 (46.0)	^d^0.562
	Yes	12 (66.7)	31 (52.5)	27 (54.0)	
Nausea; n (%)	None	0 (0)	11 (18.6)	8 (16.0)	^d^0.147
	Yes	18 (100)	48 (81.4)	42 (84.0)	
Vomiting; n (%)	None	10 (55.6)	31 (52.5)	24 (48.0)	^d^0.825
	Yes	8 (44.4)	28 (47.5)	26 (52.0)	
Photophobia; n (%)	None	5 (27.8)	8 (13.6)	4 (8.0)	^d^0.107
	Yes	13 (72.2)	51 (86.4)	46 (92.0)	
Phonophobia; n (%)	None	5 (27.8)	5 (8.5)	8 (16.0)	^d^0.108
	Yes	13 (72.2)	54 (91.5)	42 (84.0)	

SD: standard deviation; ^d^: Pearson chi-square test; ^e^: Kruskal-Wallis test; *: p < 0.05; **: p < 0.01.

A statistically significant difference between vitamin B12 measurements was found when comparing groups according to migraine frequency (patients with infrequent episodic and frequent episodic migraine were included in a single group, while patients with chronic migraine were taken as a separate group) (p = 0.011). Paired comparisons revealed lower vitamin B12 measurements in patients with chronic migraine than in the control group (p = 0.012) (Table 2). 

**Table 2.** Comparison of B12 levels according to migraine frequency and control groups.

		Migraine frequency		Control (n = 45)	p
		Episodic (n = 77)	Chronic (n = 50)		
Vitamin B12		Min-Max (median)	90-836 (227)	85-406 (184.5)	98-782 (223)	0.011*
		Mean ± SD	246.65 ± 118.88	197.50 ± 69.16	278.44 ± 149.83	
Vitamin B12 level; n (%)	< 300	55 (71.4)	45 (90.0)	28 (62.2)	^d^0.006**
	≥ 300	22 (28.6)	5 (10.0)	17 (37.8)	

SD: standard deviation; ^d^: Pearson chi-square test; ^e^: Kruskal-Wallis test; *: p < 0.05; **: p < 0.01.

Binary logistic regression analysis was done in order to investigate the effects of the vitamin B12 level (whether below or over 300), duration of the disease and presence or absence of aura on the frequency of pain. Logistic regression analysis showed a Nagelkerke ratio of 0.215 and a Hosmer-Lemeshow result of p = 0.643. We therefore came to the conclusion that the model that had been established was a good fit for the data. From the classification table, we determined that the logistic function was making classifications that were 66.9% correct. We also came to the conclusion that disease duration and vitamin B12 levels were independent factors for ‘chronic migraine’. Low vitamin B12 levels increased the likelihood of chronic migraine 3.6-fold (p = 0.022; OR = 3.687; 95% CI 1.212-11.220). In addition, the disease duration was found to be 6% effective on chronic migraine (p = 0.009; OR = 1.063; 95% CI 1.016-1.113). 

## DISCUSSION

The main conclusion from our study was that serum vitamin B12 levels in migraineurs during migraine attack-free periods were lower than the levels in the healthy control group. These levels were also lower in patients with chronic migraine than in the episodic group. Comparison of vitamin B12 levels between migraineurs without aura and the controls did not show any significant difference^13^. There was a functional vitamin B12 deficiency, represented by elevated urine MMA levels, in patients presenting migraine without aura^13^. 

Bottini et al. found that vitamin B12 levels were normal in migraineurs^10^. However, in the studies by Nelson et al. and Acar et al., like in our study, vitamin B12 levels were found to be low^11^ ^,^ ^12^_._ Fenech et al. reported that DNA damage occurred in blood cells in patients with serum vitamin B12 levels < 300 pmol/L, and that, even if serum levels were normal, vitamin B12 deficiency could occur at a cellular level. They also reported that vitamin B12 levels should be kept at > 300 pmol/L^24^. Our results also identified the need to determine vitamin B12 deficiency in patients with migraine, and supported administration of vitamin B12 to decrease pain frequency.

Causes such as neurogenic inflammation, trigeminovascular system activation, vascular dysfunction and NO release, and increased release of homocysteine (Hcy), might be responsible for the onset of migraine. Hyperhomocysteinemia, NO release and vascular dysfunction are important pathways that play a role in the pathogenesis of migraine^25^. NO-related pain transmission, hyperalgesia, chronic pain, inflammation, central sensitization and cyclic guanosine play predominant roles in monophosphate-dependent pathways. It has been suggested that vitamin B12 has a regulatory effect on inflammation and that pro-inflammatory cytokine levels become increased in patients with vitamin B12 deficiency^26^. 

Homocysteine may be responsible for endothelial dysfunction in migraine^19^. Homocysteine causes endothelial damage through NO release. Low vitamin B12 levels are correlated with high homocysteine, which in turn triggers migraine^27^. A high homocysteine level has been correlated with B12 folate and, to a lesser degree, vitamin B6 deficiency^28^. These vitamins are quite important in inhibiting hyperhomocysteinemia. Van der Kuy et al. suggested that administration of intranasal hydroxocobalamin reduced the attack frequency. Vitamin B12 plays the role of a radical scavenger against NO. Accordingly, they recommended its use for migraine prophylaxis^9^. In another study, serum vitamin B12 and methylmalonic acid (MMA) levels in patients with migraine were compared with controls. Vitamin B12 levels were found to be significantly lower in patients with migraine. A greater proportion of migraine was found in patients having low vitamin B12 levels and high MMA levels^21^.

A study conducted among patients with migraine accompanied by aura reported that vitamin supplementation (B6, B9 and B12) both significantly lowered homocysteine levels and reduced pain severity and disability, compared with placebo^16^. In another study, 51 patients with migraine with and without aura were evaluated, and decreased levels were found compared with controls. Additionally, by comparing patients with migraine during attacks and during periods without attack, it was found that vitamin B12 levels were lower during attacks. This was explained as induction of inflammation parameters by vitamin B12^12^. In that study, migraines were classified as aura or non-aura, but no remarks were offered regarding pain frequency^12^. All vitamin B12 samples from the cases included in our study were taken at times in between the attacks. We also found in our study that vitamin B12 levels were lower in cases with higher pain frequency.

In addition, we investigated the correlation between B12 levels and migraine pain frequency and severity. We found that vitamin B12 levels were lower in the chronic migraine group, which had greater frequency and severity of pain. Patients with chronic migraine are given analgesics that are more powerful, because of their pain. One possible cause of vitamin deficiency may be long-term and voluminous use of non-steroidal anti-inflammatory drug (NSAID) medication, thereby causing pathological conditions of the gastrointestinal system, which in turn disrupt vitamin absorption^14^. In our study, when patients with migraine were classified into subgroups, the vitamin B12 levels of patients with chronic migraine were found to be significantly lower than those of patients with infrequent and frequent episodic migraine. There was no significant difference between patients with frequent episodic and chronic migraine. Patients with infrequent and frequent episodic migraine were also similar. The vitamin B12 deficiency in chronic migraine cases may be caused by frequent use of analgesics. Prospective studies assessing analgesic use and vitamin B12 levels may be interesting. Use of analgesics was not taken into consideration in our present study.

Vitamin B12 and folic acid levels have been found to be lower in children with migraine than in normal controls^20^. Decreased frequency of migraine headache and improved hyperhomocysteinemia were reported after folic acid treatment was administered to 16 children with migraine, hyperhomocysteinemia and MTHFR gene mutations, followed by a three-month follow-up. From that result, it was recommended that children with hyperhomocysteinemia should be administered folic acid for migraine prophylaxis^17^. In the same way as for adults, vitamin B12 is also recommended in prophylactic treatment for pediatric and adolescent patients^10^. Our patient group consisted of patients aged over 18 years. The results from our study were comparable to those from studies assessing child populations.

Several studies have been demonstrating a relationship between migraine and nutritional deficiencies. In one study, vitamin D levels were found to be low in patients with migraine^29^. Low magnesium levels were reported in patients with migraine, especially during an attack^30^. Metabolic boosters such as riboflavin and coenzyme Q10 have been used to treat ketogenic migraine through diet, or pharmacologically^31^. In migraine, it is important to consider nutritional supplements from a wider perspective. Evaluating these parameters is important in terms of general health and preventive medicine. We only assessed vitamin B12 levels in our cases. We did not assess other nutritional deficiencies.

The limitation of our study was that it was a cross-sectional retrospective study conducted at a tertiary care training facility. This group reflects a small portion of a large migraine population. Due to the nature of our institution as a tertiary care center, the majority of our cases comprised persistent frequent episodic migraine and chronic migration. Another limitation of the study was that folic acid, homocysteine and methylmalonic acid levels were not measured along with vitamin B12. Planning a study as multicentered, and for a larger patient group, may yield more useful results.

In conclusion, there is a negative correlation between migraine and vitamin B12 levels. In our study, we compared vitamin B12 values according to frequency of pain. We found lower levels in the chronic migraine group. When planning treatments for these patients, it is important to take a holistic approach when considering complementary treatments. There are many arguments in favor of the importance of B12 in cases of migraine with aura. However, more randomized-controlled studies are needed in relation to migraine without aura. With these data, controlled replacement treatment planning seems to be important. Controlled studies are needed in order to determine the direction to which the frequency and intensity of pain will tend when vitamin B12 is replaced in chronic migraine.
